# Mandibular myofibrosarcoma of childhood: Surgical resection & reconstruction with fibula flap

**DOI:** 10.1590/S1808-86942011000300024

**Published:** 2015-10-19

**Authors:** Konstantinos Kourelis, Yelizaveta Shnayder, Vincent Key, Douglas Girod, Terrance Tsue

**Affiliations:** 1 MD, MSc, PhD (Fellow, Kansas University Medical Center, Department of Otolaryngology); 2 MD, FACS (Assistant Professor, Kansas University Medical Center, Department of Otolaryngology); 3 MD, FACS (Assistant Professor, Kansas University Medical Center, Department of Orthopedic Surgery); 4 MD, FACS (Professor, Kansas University Medical Center, Department of Otolaryngology); 5 MD, FACS (Professor, Kansas University Medical Center, Department of Otolaryngology); Kansas University Medical Center

**Keywords:** mandible, sarcoma, surgical flaps

## INTRODUCTION

Myofibrosarcomas are rare soft-tissue sarcomas mainly affecting the head and neck or extremities of adult patients^1^. The evolution of the cell of origin, that is the myofibroblast, still eludes us. Described as a modified common fibroblast, it is either a native constituent of normal tissues, or the result of tissue reactive processes.

The natural course of myofibroblastic malignancies follows a slowly-growing, infiltrative pattern of spread, but carries a risk of recurrence and metastasis, even after many years. The latter attribute can be explained by the lack of a capsule^2^.

Below, we report a case of a mandibular myofibrosarcoma in an 11-year-old girl.

## CASE PRESENTATION

An 11-year-old girl presented with a left-sided mandibular tumor. Initially, it had been enucleated transorally, before the patient was evaluated in our clinic. Histopathology was consistent with intermediate-to-high grade myofibrosarcoma. A CT scan one month post-operatively demonstrated a residual mass, measuring 3.7cm transverse × 1.9cm anteroposterior × 3.4cm cephalo-caudal, and involving the majority of the ramus.

**Figure 1:**
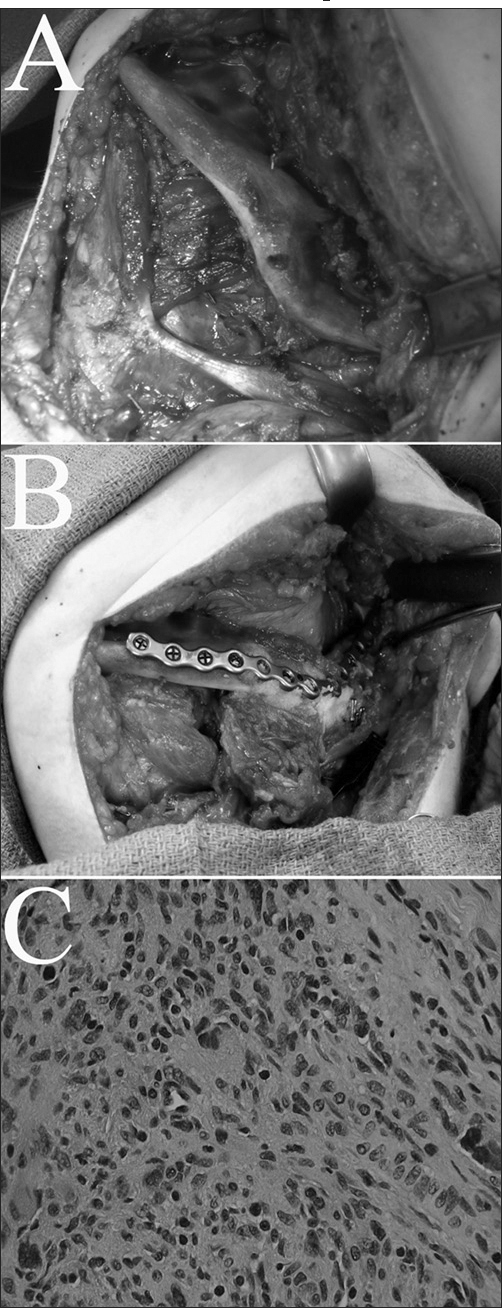
(A) Intraoperative photograph before resection, demonstrating erosion of the angle of the mandible by the tumor. (B) The osteotomized fibula bone is inset within the mandibular defect, and anchored to a titanium plate. (C) Microphotograph of the excised myofibrosarcoma. The typical morphology of the myofibroblasts comprises an elongated, eosinophilic cytoplasm surrounding a denser indented nucleus, and is a combination of a smooth muscle cell and a fibroblast. (Staining: hematoxylin and eosin. Original magnification: x400)

Segmental mandibulectomy was performed, encompassing the angle of the jaw(Fig. 1A). An osseofasciocutaneous fibula flap was harvested, including 10cm of bone. The straight fibular segment, prior to its incorporation into the defect was properly osteotomized to resemble the shape of the jaw angle(Fig. 1B). The soft-tissue portion of the flap was inset intraorally to cover the fibular bone. In addition, it would be used as a monitoring paddle. Before closure of the donor site, orthopedic surgeons performed osteosynthesis of the distal fibula to the tibia, to secure the stability of the ankle.

The pathology report was consistent with myofibrosarcoma(Fig. 1C). Six months after the procedure, there is no evidence of recurrence or metastasis. In addition, the patient demonstrates acceptable occlusion and speech quality. No donor site complications were noted, and the child gradually resumes the physical activities of her peer group.

## DISCUSSION

Malignancies showing myofibroblastic differentiation are very rare and their biologic behavior is far from being elucidated. Myofibrosarcomas are soft-tissue sarcomas but a few of them are intraosseous, as the case with our patient.

Myofibroblasts(Fig. 1C), which give rise to these sarcomas, are reactive cell types, induced by trauma, fibrosis and adjacent tumors. None of the above situations was noted in our patient. Interestingly, the normal periodontal ligament is among the very few sites in which a native myofibroblastic population has been detected^2^, but the nidus of our patient's neoplasm was posterior to the tooth-bearing mandible.

11 cases of myofibrosarcoma of the head and neck have been reported in children. Myofibrosarcomas respond poorly to chemotherapy and radiation therapy^3^.

In the pediatric population, reconstruction following surgical resection is challenging, both in terms of donor site morbidity and rebuilding the growing facial skeleton. With regard to complications developing in the donor site, ankle instability is reported in 27% of children only, due to their greater ligamentous laxity^4^. As this deformity might become permanent in adulthood, the authors suggest prophylactic tibiofibular synostosis with screws, which was performed by our orthopedic team in this case.

The female mandible has achieved 90% of the adult size at the age of 11^5^. Segmental replacement with a living tissue (fibular bone) and a synthetic (plate) component carries the risk of esthetic asymmetry and functional malocclusion. Whereas plates do not interfere with mandibular growth, they are eventually surrounded by neobone. The encased hardware weakens the bone stock and poses challenges to future mandibular surgery^6^. Consequently, we could consider plate removal at a later age.

## FINAL COMMENTS

Myofibrosarcoma is a rare and undefined entity, so that every new report of a case might contribute to understanding its natural course and management. In view of all the previous reports, the treatment of choice should be surgical resection. Here, we presented a case in a pediatric patient, trying to emphasize the importance of a comprehensive approach to a superior oncological outcome, as well as acceptable quality of life.
